# Advances in Neuroinflammation

**DOI:** 10.3390/brainsci14100965

**Published:** 2024-09-26

**Authors:** Junhui Wang, Jing Sun, Hongxing Wang

**Affiliations:** 1 Lunenfeld-Tanenbaum Research Institute, Mount Sinai Hospital, Toronto, ON M5G 1X5, Canada; 2 Thyropathy Hospital, Sun Simiao Hospital, Beijing University of Chinese Medicine, Tongchuan 727000, China; 3 Department of Pathology, Capital Medical University, Beijing 100069, China; sunjing@ccmu.edu.cn; 4 Department of Neurology, Xuanwu Hospital, Capital Medical University, Beijing 100053, China; wanghongxing@xwh.ccmu.edu.cn

Recent research in neuroscience has shown significant advancements in relation to neuroinflammation, especially its role in neurological diseases, including neurodegenerative diseases. Over-activated central nerve system (CNS) “immune cells”—astrocytes and microglial cells with their subsequent cytokines and chemokines—are, *sine qua non*, fundamental mechanisms of pathogenesis in many neurological diseases [1]. Neuroinflammation acts as a double-edged sword in the CNS. While working as a defense response against brain insults by removing toxic agents and minimizing the detrimental effects, a prolonged battle in the process of inflammation will result in the development of chronic neuroinflammatory conditions, such as neurodegenerative diseases [2]. Therefore, we are facing a dilemma in pinpointing the role of neuroinflammation in the pathological or even physiological processes of the CNS. The primary goal of this Special Issue, titled “Advances in Neuroinflammation”, is to investigate the recent advances in neuroinflammation research in both preclinical and clinical areas, in particular, but not limited to, neurodegenerative diseases. We collected and published five research and five review papers from distinguished scientists in the field; each publication addresses the role of neuroinflammation in neurological diseases from the novel perspective of the authors, presenting possible new direction for future study in the field. 

In the review papers, the authors aim to decipher the possible role and importance of neuroinflammation in CNS diseases, especially neurodegenerative diseases. For years, contradictory findings have existed concerning the association between both aberrant apolipoprotein E (APOE)-ε4 and serum lipids and the occurrence of Alzheimer’s disease (AD) [3,4]. In the first paper, Xu et al. performed a meta-analysis to investigate the relationship of apolipoprotein E alleles and serum lipids with; they found that the elevated total cholesterol (TC) and low-density lipoprotein (LDL) levels showed considerable heterogeneity between patients with AD and healthy controls. Higher TC and LDL levels were found in APOEε4 allele carriers compared with non-carriers, and the difference was more significant in patients with AD compared to healthy controls. Their results supported the hypothesis that the APOEε4 allele might lead to the development of AD through influencing lipid metabolism (Contribution 1). 

As mentioned above, neuroinflammation is closely related to the onset of many neurological diseases. However, the underlying mechanisms have not yet been fully deciphered. Balistreri et al. presented a review paper to discuss the recent progress in this field, focusing on the important roles of peripheral and infiltrated monocytes and clonotypic cells, the gut–brain axis, the apelinergic system, the endothelial glycocalyx of the endothelial component of neuronal vascular units, non-coding RNA and other types of gene expression, etc., in the development of neurological diseases. This review illustrated the complex neuroinflammatory reactions and novel mechanisms proposed by the authors, significantly adding valuable information to the comprehension of the complex etiological link between neuroinflammation and neurological diseases (Contribution 2).

AD is a detrimental CNS disease with immense complexity in terms of its mechanisms, which is a major obstacle in understanding its pathogenesis. The third paper, authorized by prestigious scientist Weaver Donald, proposed that neuroinflammation was the central player in unifying 30 different risk factors of AD; this review identified 30 risk factors for AD and extended the analysis to further identify neuroinflammation as a unifying player presented in all of these risk factors. In this review, the author claimed that the dysfunction of the neuroimmune–neuroinflammation axis was central to all 30 identified risk factors. Though the nature of the neuroinflammatory involvement varies in different conditions, the activation of glial cells and the release of pro-inflammatory proteins are common pathways shared by all these risk factors. While discussing a very novel point of view, this review article provided further evidence of the importance of neuroinflammatory mechanisms in the etiology of AD (Contribution 3). 

In another review paper, Yang et al. detailed the role of astrocytes in Amyotrophic Lateral Sclerosis (ALS) and emphasized the importance of the non-cell-autonomous role of astrocytes in this detrimental CNS disease. According to this paper, astrocytes can play a pivotal role in ALS development via participating in calcium homeostasis imbalance, mitochondrial dysfunction, abnormal lipid and lactate metabolism, glutamate excitotoxicity, etc. This review systemically outlined the possible contributions of reactive astrocytes in the pathogenesis of ALS. More importantly, comprehensive evidence is provided stating that astrocytes could be the potential therapeutic target for the treatment of ALS (Contribution 4). 

Glioblastoma, as the most common and malignant brain tumor, usually presents with high morbidity and mortality [5]. Extreme complication of the tumor microenvironment is a formidable challenge in advancing glioblastoma therapy for the medical community, and neuroinflammation is characterized by a variety of resident or infiltrating inflammatory cells—key players in creating this complexity. In their review paper, Li et al. pointed out that neuroinflammation not only builds a unique tumor environment for glioblastoma cells to develop and grow, but also played important roles in regulating tumor aggressiveness and treatment resistance; they also emphasized that the anti-tumor microenvironment interventions, such as anti-inflammation, could be used as potential therapeutic tools against glioblastoma in the future (Contribution 5).

Chronic idiopathic demyelinating polyneuropathy (CIDP) is an acquired, immune-mediated neuropathy with very limited treatment options thus far [6]. Recently, subcutaneous immunoglobulins (SCIgs) have been employed in clinical setting as a maintenance therapy for CIDP. In the first research article of this Special Issue, Alonge et al. retrospectively explored electrophysiological and efficacy data from 15 patients who received the SCIg treatment. They reported that SCIg maintenance therapy could preserve nerve function in CIDP with good efficacy and safety properties. Electronystagmography can be used to evaluate treatment effectiveness and was a useful instrument for the follow-up and prognostic assessment of CIDP. This study further strengthened evidence in relation to the efficacy of SCIg maintenance therapy in CIDP (Contribution 6). 

Sleep deprivation could adversely impact immune function, cognitive memory, learning ability, etc. Studies have revealed that sleep deprivation can lead to inflammatory responses in the CNS. Li et al. investigated the protective role of dexmedetomidine, an anesthetic compound, in sleep deprivation by focusing on its possible anti-inflammatory role in the CNS. In a sleep deprivation mouse model, the authors claimed that dexmedetomidine could significantly improve anxiety-like behaviors in the sleep-deprived mice and could attenuate inflammatory responses and oxidative stress in the CNS by inhibiting the activation of the p38/MSK1/NFκB pathway (Contribution 7). 

Epidermal growth factor receptor (EGFR) gene deficits and the subsequent activation of the EGFR signaling pathway promote the genesis of gliomas [7]. However, whether factors exist within the microenvironment that can lead to EGFR activation is currently unknown. In a clinical study on glioma, Zhou et al. probed the association between the EGFR and IFN-γ pathways and their possible synergistic effects on survival prediction and immune escape in glioma patients. Their study concluded that cytokine IFN-γ might be an upstream trigger of EGFR signaling activation, and EGFR-related and IFN-γ-related signatures could be jointly used to stratify patients into well-defined risk groups. High-risk patients tended to have a poorer prognosis and a more inhibitory microenvironment. They claimed that these patients might be more suitable for immune checkpoint blockade (ICB) therapy or other immunotherapeutic approaches (Contribution 8). 

In a clinical study of insomnia, Wang et al. investigate the clinical efficacy of biofeedback on insomnia and its potential neural mechanisms. They found that a biofeedback treatment based on alpha power and prefrontal EMG could relieve insomnia and ameliorate anxiety and depression. The underlying mechanism may be attribute to increased alpha power, decreased beta and theta power, and decreased EMG power (Contribution 9).

The last study in this Special Issue, authored by Li et al., used a mendelian randomization (MR) method to investigate the causal effect of circulating inflammatory proteins on multiple sclerosis (MS) by digging into the data from a large-scale genome-wide association study (GWAS). They reported that 91 circulating inflammatory proteins were closely associated with the onset and progression of MS. They claimed that this study provided new insights into the relationship between circulating inflammatory proteins and MS, and discussed the possibility of using these circulating inflammatory proteins as potential biomarkers and therapeutic targets for MS in the future (Contribution 10).

In a nutshell, the above studies in our Special Issue pinpoint the importance and complexity of neuroinflammation from multiple perspectives, and strengthen the idea that neuroinflammation plays a very important role in a variety of neurological diseases, especially neurodegenerative diseases. In general, our Special Issue highlights the urgency to further investigate the inflammatory role in disease genesis with continuous research by using diverse new tools and technologies. 
